# Burnout in Australian sport and exercise physicians and registrars: A cross-sectional study

**DOI:** 10.1016/j.jsampl.2024.100074

**Published:** 2024-08-14

**Authors:** Bikram Karmakar, Ping-I Lin, Hindol Mukherjee, James Rufus John, Valsamma Eapen

**Affiliations:** a Sport and Exercise Physician, Adjunct Associate Lecturer UNSW School of Medicine, Balmain Sports Medicine, Australia; b Discipline of Psychiatry and Mental Health, School of Clinical Medicine, University of New South Wales, Australia; c Department of Psychiatry and Behavioral Neuroscience, School of Medicine, Saint Louis University, USA; d Northern Beaches Hospital, Australia; e BestSTART-SWS, South Western Sydney Local Health District, School of Clinical Medicine, UNSW Medicine & Health, Australia; f Infant Child and Adolescent Psychiatry (Department of Psychiatry), University of New South Wales, Sydney, Australia

**Keywords:** Sports medicine, Occupational health, Stress, Career burnout, Mental health

## Abstract

**Objectives:**

To determine the prevalence and factors associated with the risk of burnout among sport and exercise physicians and registrars in Australia.

**Design:**

Cross-Sectional Study.

**Method:**

Australian based sport and exercise physicians and registrars of the Australasian college of sport and exercise physicians were invited to complete a survey. Demographic data and response to a questionnaire utilising the Maslach Burnout Inventory (MBI) tool were collected. Descriptive analysis was conducted to assess the prevalence of burnout. Multivariable logistic regression models were used to determine factors associated with the risk of burnout whilst adjusting for covariates.

**Results:**

25 registrars (25/52 ​= ​48%) and 31 physicians (31/172 ​= ​18%) completed the survey. The risk of overall burnout in all three MBI parameters or in at least one parameter was 25% and 34%, respectively. Findings of the primary analysis showed that higher working hours (AOR 50.59; 95% CI 1.81–141.33; p ​= ​0.021) and higher level of job dissatisfaction (AOR 262.68; 95% CI 4.98–13857.50; p ​= ​0.006) were associated with increased odds of burnout.

**Conclusions:**

Burnout poses a significant risk to sport and exercise physicians and registrars in Australia. The small size of this specialty group and unique nature of their work requires specific interventions to reduce risks of burnout.

## 1 Introduction

Burnout has been defined as a psychological syndrome consisting of emotional exhaustion, depersonalisation, and reduced personal accomplishment [1]. Burnout in physicians is estimated to range between 0 and 85%, globally [2]. The wide variation in prevalence rates may arise from heterogeneity in ascertainment methods, cultural differences across populations, and differences in workplace regulations. The physical and emotional exhaustion experienced by healthcare professionals can lead to decreased job satisfaction, increased rates of medical errors, and impaired decision-making. Further, burnout can contribute to higher rates of absenteeism, staff turnover, and overall dissatisfaction within the healthcare industry, exacerbating the existing workforce shortage and compromising patient outcomes [3].

One of the widely used measures of burnout is the Maslach Burnout Inventory (MBI), which is a validated assessment tool used to assess the prevalence and characteristics of burnout in physicians [2]. The MBI assessment tool divides burnout into three dimensions: exhaustion, depersonalisation, and reduced personal accomplishment [1]. These categories exhibit strong correlation with medical student, physician, and surgeon well-being measures [4]. In physician assessment studies, various definitions of overall burnout have been used. Overall burnout using the MBI scale has most often been defined when exhaustion and depersonalisation have scored in the high range and/or personal satisfaction has scored in the low range [2].

Other factors have been demonstrated to impact on burnout seen in healthcare workforce studies. Significant research has undertaken to explore personality traits to determine their impact on burnout rates. Recent studies have suggested that personality trait assessment may assist in predicting and intervening early in those at risk for burnout syndrome [5]. Long working hours has been demonstrated to be a significant factor associated with burnout in public hospitals and subjective well-being [6]. Gender differences often exist in physician work life [7]. This correlates with higher burnout rates in women and indicate that specific interventions are likely required to intervene in this group [8].

Interest and awareness of burnout has been increasing exponentially. The number of PubMed hits for the search “burnout” rose from 27 in 1980–2145 in 2019 [9]. However, the incidence of burnout has not been systematically investigated in sport and exercise physicians. Sport and exercise physicians often hold multiple positions, including clinic-based roles, field work with sport groups, travelling work as a tour doctor, and administrative roles developing healthcare policy for sporting organisations. Sport and exercise physicians often find themselves as the medical leader in many, if not all their roles. Their responsibility often exceeds what may have been initially anticipated. Attending to other duties on weekends and out of clinic time may be a specific factor leading to burnout in sports physicians. This has been likened to "Athlete Overtraining" whereby excess workload eventually leads to reduction in performance [10]. This study aimed to determine the prevalence and factors associated with the risk of burnout in sport and exercise physicians and registrars in Australia.

## 2 Methods

Ethics approval was attained from the UNSW Sydney Institutional Human Research Ethics Committee (HC210927). A survey was sent via email to Australian based sport and exercise physicians and registrars as identified by the Australasian College of Sport and Exercise Physicians. Information about the aim of the study was provided to participants in an information sheet along with the consent form. No identifying information including names of participants was collected. Demographic data including current role (physician or registrar), gender (male, female, non-binary), age (in years), previous psychiatric history, and years of experience were collected. All surveys were completed between May and October 2022.

Primary outcomes were measured using the 22-item Maslach Burnout Inventory tool with 3 subscale scores measured; A: exhaustion (7-items), B: depersonalisation (7-items), C: reduced personal achievement (8-items). Each subscale was scored individually. Burnout in one category occurred if the following scores occurred in each category: ≥30 for Section A or ≥12 for Section B or ≤33 for Section C. Overall burnout was defined as high scores in A ​+ ​B, A ​+ ​B ​+ ​C, or C alone. Additionally, there were two questions to assess satisfaction of career choice and work-life balance which was assessed using a 5-point Likert scale.

Descriptive statistics was conducted to ascertain the baseline characteristics of the sample and reported as frequency counts with percentages. Association between categorical variables of ‘overall burnout’ or ‘no overall burnout’ were compared using Fisher's exact test. Correlation analysis was used to assess the strength of the association between the 3 subscales of the MBI tool. For the primary analysis, multivariable regression models were performed to determine the significant predictors associated with the risk of burnout (model 1 – overall burnout and model 2 – burnout in at least one parameter), whilst adjusting for sociodemographic covariates. A p-value <0.05 was defined as statistically significant.

## 3 Results

A total of 56 participants out of the 224 Australian based sport and exercise physicians and registrars responded, giving a response rate of 25%. The response rate was higher amongst registrars (25/52 ​= ​48%) compared to physicians (31/172 ​= ​18%). Overall burnout was evidenced in 25% of all responders while burnout in at least one of the 3 Maslach Parameters was observed in 34% of the sample.

The characteristics of the participants are shown in Table 1. Of the responders, 69.6% identified as male with the remaining 30.4% as female. Only one responder identified as Aboriginal or Torres Strait Islander. Participant characteristics did not significantly differ among those who reported burnout compared to those who did not report burnout, except for job satisfaction. There were no differences in burnout risk between physicians and registrars. Those who answered ‘not sure’ to having job satisfaction had high risk of having overall burnout (p ​= ​0.008).

**Table 1:** Descriptive analysis for baseline characteristics based on overall burnout (Fisher's exact test).

Variable	Response number (%)	Overall burnout		p-value
		No N (%)	Yes N (%)	
**Age**				0.610
*19-29 years*	2 (3.6)	2 (100.0)	0 (0.0)	
*30-39 years*	21 (37.5)	14 (66.7)	7 (33.3)	
*40-49 years*	12 (21.4)	8 (66.7)	4 (33.3)	
*50-59 years*	15 (26.8)	13 (86.7)	2 (13.3)	
*60 years and above*	6 (10.7)	5 (75.0)	1 (25.0)	
**Gender**				1.000
*Male*	39 (69.6)	29 (74.4)	10 (25.6)	
*Female*	17 (30.4)	13 (76.5)	4 (23.5)	
**Aboriginal or Torres Islander status**				N/A
*No*	55 (98.2)	41 (74.5)	14 (25.5)	
*Yes*	1 (1.8)	1 (100.0)	0 (0.0)	
**Current job title**				0.759
*Sports and Exercise Registrar*	25 (44.6)	18 (72.0)	7 (28.0)	
*Sports and Exercise Physician*	31 (55.4)	24 (77.4)	7 (22.6)	
**Work experience**				0.501
*0-5 years*	2 (3.6)	1 (50.0)	1 (50.0)	
*6-10 years*	17 (30.4)	11 (64.7)	6 (35.3)	
*11-15 years*	11 (19.6)	9 (81.8)	2 (18.2)	
*More than 15 years*	26 (46.4)	21 (80.8)	5 (19.2)	
**Average hours worked per week**				0.730
*0-20 ​h*	1 (1.8)	1 (100.0)	0 (0.0)	
*20-30 ​h*	6 (10.7)	4 (66.7)	2 (33.3)	
*30-40 ​h*	9 (16.1)	8 (88.9)	1 (11.1)	
*40-50 ​h*	23 (41.1)	18 (78.3)	5 (21.7)	
*50-60 ​h*	10 (17.9)	6 (60.0)	4 (40.0)	
*More than 60 ​h*	7 (12.5)	5 (71.4)	2 (28.6)	
**State where most worked from**				0.950
*New South Wales*	21 (37.5)	15 (71.4)	6 (28.6)	
*Victoria*	16 (28.6)	11 (68.8)	5 (31.3)	
*Queensland*	8 (14.3)	6 (75.0)	2 (25.0)	
*Western Australia*	4 (7.1)	4 (100.0)	0 (0.0)	
*Australian Capital Territory*	4 (7.1)	3 (75.0)	1 (25.0)	
*Tasmania*	2 (3.6)	2 (100.0)	0 (0.0)	
*Northern Territory*	1 (1.8)	1 (100.0)	0 (0.0)	
**Marital status**				
*Married*	39 (69.6)	29 (74.4)	10 (25.6)	
*Defacto or partner but not married*	12 (21.4)	10 (83.3)	2 (16.7)	
*Single*	5 (8.9)	3 (60.0)	2 (40.0)	
**Past psychiatric diagnosis**				0.698
*No*	46 (82.1)	35 (76.1)	11 (23.9)	
*Yes*	10 (17.9)	7 (70.0)	3 (3.0)	
**Type of psychiatric diagnosis**				
*Depression*	5 (8.9)	4 (80.0)	1 (20.0)	1.000
*Anxiety*	6 (10.7)	4 (66.7)	2 (33.3)	0.633
*Bipolar disorder*	1 (1.8)	1 (100.0)	0 (0.0)	1.000
*Post-traumatic stress disorder*	0 (0.0)	0 (0.0)	0 (0.0)	–
*Schizophrenia*	0 (0.0)	0 (0.0)	0 (0.0)	–
**Job satisfaction**				0.008
*Very dissatisfied*	0 (0.0)	0 (0.0)	0 (0.0)	
*Moderately dissatisfied*	4 (7.1)	3 (75.0)	1 (25.0)	
*Not sure*	5 (8.9)	1 (20.0)	4 (80.0)	
*Moderately satisfied*	24 (42.9)	17 (70.8)	7 (29.2)	
*Very satisfied*	23 (41.1)	21 (91.3)	2 (8.7)	
**Work-life balance**				0.122
*Very dissatisfied*	4 (7.1)	1 (25.0)	3 (75.0)	
*Moderately dissatisfied*	16 (28.6)	11 (68.8)	5 (31.3)	
*Not sure*	5 (8.9)	5 (100.0)	0 (0.0)	
*Moderately satisfied*	24 (42.9)	19 (79.2)	5 (20.8)	
*Very satisfied*	7 (12.5)	6 (85.7)	1 (14.3)	

Findings of the Pearson's correlation analysis showed significantly high positive correlation between exhaustion scores and depersonalisation scores (Table 2). Additionally, depersonalisation scores were moderately inversely correlated with reduced personal achievement scores. Cronbach's alpha was 0.838, indicating a high level of internal consistency for our scale with this specific sample.

**Table 2:** Correlation of subscales of burnout.

Correlation	Exhaustion score	Depersonalisation score	Reduced personal achievement score
Exhaustion score	1	0.696∗∗	−0.162
Depersonalisation score	0.696∗∗	1	−0.338∗
Reduced personal achievement score	−0.162	−0.338∗	1

∗∗p-value<0.01, ∗p-value<0.05.

In the primary analysis, findings of the multivariable logistic regression analyses (Table 3) showed that those who reported working for more than 50 ​h per week had significantly higher odds of both overall burnout (AOR 11.31; 95% CI 0.91–140.36; model 1) and burnout in at least one parameter (AOR 50.59; 95% CI 1.81–141.33; model 2) compared to working less than 40 ​h per week. Furthermore, compared to those who reported unsatisfactory levels of job satisfaction, participants who reported unsure or unsatisfactory levels of job satisfaction had significantly higher odds of burnout in both models (model 1: AOR 17.16; 95% CI 1.52–194.37), model 2: AOR 262.68; 95% CI 4.98–13857.50)). Participants who were in de facto relationships or who had partners but were not married had a moderate but significant association with burnout in model 1 but not model 2.

**Table 3:** Multivariate logistic regression analysis.

Variable	Model 1 Outcome measure - Overall burnout	Model 2 Outcome measure -Burnout in at least one parameter
	AOR with 95% CI [p-value]	AOR with 95% CI [p-value]
**Age**		
*19-49 years*	Reference category	Reference category
*50 years and above*	0.33 (0.05–2.35)	0.32 (0.04–2.79)
**Gender**		
*Male*	Reference category	Reference category
*Female*	0.95 (0.19–4.78)	1.64 (0.24–11.13)
**Current job title**		
*Sports and Exercise Registrar*	Reference category	Reference category
*Sports and Exercise Physician*	1.73 (0.32–9.25)	3.09 (0.42–22.83)
**Average hours worked per week**		
*0-40 ​h*	Reference category	Reference category
*40-50 ​h*	2.07 (0.32–13.45)	5.39 (0.48–61.13)
*More than 50 ​h*	11.31 (0.91–140.36)∗ [0.05]	50.59 (1.81–141.33)∗ [0.021]
**Marital status**		
*Married*	Reference category	Reference category
*Defacto or partner but not married*	0.23 (0.02–2.39)	0.01 (0.01–0.48)∗ [0.002]
*Single*	2.28 (0.27–19.57)	3.94 (0.36–43.02)
**Past psychiatric diagnosis**		
*No*	Reference category	Reference category
*Yes*	1.47 (0.25–8.81)	6.36 (0.81–50.11)
**Job satisfaction**		
*Satisfactory*	Reference category	Reference category
*Unsatisfactory/unsure*	17.16 (1.52–194.37)∗ [0.022]	262.68 (4.98–13857.50)∗ [0.006]
**Work-life balance**		
*Satisfactory*	Reference category	Reference category
*Unsatisfactory/unsure*	0.24 (0.03–2.28)	0.18 (0.01–2.47)

AOR – Adjusted odds ratio; ∗p-value<0.05.

The variable ‘work experience’ was removed due to its collinearity with other variables.

## 4 Discussion

To the best of our knowledge, no published studies have investigated burnout exclusively in sport and exercise medical practitioners. Sport and exercise physicians in Australia often hold multiple positions in clinical and non-clinical roles and typically work outside the hospital system. The rate of burnout seen in hospital-based doctors is unlikely to be representative of the work of the sport and exercise specialty cohort.

The rate of overall burnout in this study is lower than the rates identified in other health professional groups in Australia. For example, burnout in Australian orthopaedic trainees has been measured at 53% [11]. Our study did not indicate any significant difference in burnout rate between physicians and registrars in training while previous studies have reported such differences based on years of experience [12,13]. A study of US physicians found higher prevalence of overall burnout (37.9%) compared to the general population (29.8%) and were more likely to have high risk of at least one category of burnout [12]. Further, it is interesting to note that among US physicians, professional burnout has changed over time with at least one category positive in 45.5% in 2011 compared to 54.4% in 2014 and 43.9% in 2017 [14]. The wide range of burnout rates suggests that further studies in the same and comparative population groups are required to evaluate trends and understand contextual factors that may account for differences.

### 4.1 Implications of the research

Burnout continues to pose a significant threat to the medical workforce. There are several factors which are associated with increased rates of burnout amongst healthcare professionals and specifically physicians. There is a dose–response association between increased burnout and incremental increase in hours worked per week [15]. Similarly, perceived increases in case load and decreased levels of autonomy in practice amongst physicians, has been associated with increased levels of global burnout and physical fatigue [16]. In our study, depersonalisation, and emotional exhaustion on the MBI tool appeared to correlate closely with increased rates of burnout in the sport and exercise cohort. Physician burnout has been associated with other systemic factors including inefficient work processes, clerical burdens, leadership culture and conflicts associated with work-home balance [3]. Thus, evaluating variables of burnout in sport and exercise physicians, allow comparison with existing research which in turn may provide crucial information on systemic or individual changes necessary to reduce levels of burnout.

Unaddressed burnout in healthcare workers leaves a significant economic dent. Physician turnover and reduced hours are two factors associated with burnout that are estimated to cost $4.6 billion to the US health industry [17]. Correlation between single-item measures of the MBI and outcomes including dropout, medical error and suicidality has been previously demonstrated [4]. Sport and exercise physicians and registrars are a small cohort, and the implications of any burnout are likely to be significant. Solutions to combat healthcare burnout are best applied when understanding variables in specific specialty groups. Correlation with long working weeks indicates that this is an important variable to be addressed while association with relationship status requires further exploration. Specific strategies to prioritise life decisions and work life balance for sports physicians include developing mentorship, improving work efficiency, and fostering growth and support in the workplace [10].

Differing demands and accountability may be placed on a consultant compared to a trainee. The difference in the risk of burnout between registrars and specialists was not significant in this paper due to its relatively smaller effect size compared with other variables, such as working hours and job satisfaction, judged from the point estimates in odds ratios. The point estimates for odds ratios corresponding to the job title fell between 1.73 and 3.09, implying that the significance might be endorsed in a larger sample. Therefore, the current study might underestimate the higher risk of burnout in trainees than in specialists. Similarly, a difference in burnout rates could not be established between male and female sport and exercise medicine doctors in this sample. The disproportionate number of female responders reflects the under-representation of females that has been previously described in sport and exercise medicine [18]. Though this paper could not establish burnout as a factor for this, it suggests that further research into gender disparity in sport and exercise medicine is necessary.

### 4.2 Limitations

There were several limitations to this study. Firstly, as there are no standardised diagnostic criteria for Burnout, the features may overlap with depression, chronic fatigue syndrome and other conditions. The Maslach Burnout Index used in this study is a tool to assist identification of burnout. Hence it may be more accurate to classify those with positive findings on this scale as having ‘high risk of burnout’ rather than having burnout. Secondly, the total number of registrars and physicians in the Australasian college of sport and exercise is significantly lower than most other specialties. The survey response rates, while comparable to other similar study designs, had a low sample size due to the smaller base population from which this was drawn. The estimated prevalence rate is likely to have a wide confidence interval, leading to inaccurate prevalence estimates in the population. Additionally, a small sample size increases the likelihood of sampling variability, which refers to the natural variation in estimates that occurs due to random sampling. This variability can lead to inconsistent estimates across different samples and make it challenging to determine the true prevalence. This can also lead to selection bias; however, we were unable to compare characteristics between survey respondents and non-respondents to determine how this may impact findings. Given the small sample size in this study, it is recommended to select variables based on existing literature rather than solely relying on statistical criteria (such as p-values from univariate analyses) due to the increased risk of overfitting, inflated Type I error rates, and unreliable estimation of parameters [19]. Future studies do require collective efforts amongst members of this cohort to increase response rates.

## 5 Conclusion

The risk of ‘overall burnout’ in sport and exercise physicians and registrars was 25% while at least one parameter for burnout was evidenced in 34% of participants. Longer working hours were associated with a higher risk of burnout in this speciality group. The rate of burnout is slightly lower than other specialty groups. As the sport and exercise physician community in Australia is smaller than other medical speciality organisations, interventions to combat burnout at an organisational and individual level should be specific and prioritised to reduce future risk.

## Ethical compliance

Ethical compliance has been made. Ethics approval was attained from the UNSW Sydney Institutional Human Research Ethics Committee (HC210927)

## Funding statement

This research did not receive any specific grant from funding agencies in the public, commercial, or not-for-profit sectors.

## Declaration of competing interest

The authors declare that they have no known competing financial interests or personal relationships that could have appeared to influence the work reported in this paper.
